# Colon Capsule Endoscopy-Measured Colonic Transit Time Is Associated with Frailty in Older Adults

**DOI:** 10.3390/medicina62071362

**Published:** 2026-07-15

**Authors:** Konosuke Nakaji, Mitsutaka Kumamoto, Yukinori Nakae

**Affiliations:** Endoscopy Center, Aishinkai Nakae Hospital, 30-1 Funadokoro, Wakayama-shi 640-8461, Wakayama, Japan; kuma_mitsu@nakae.or.jp (M.K.); nakae12345@gmail.com (Y.N.)

**Keywords:** clinical frailty scale, colonic transit time, frailty, older adults, colon capsule endoscopy, constipation, sex differences

## Abstract

*Background and Objectives*: Although the association between frailty and constipation in older adults has attracted growing attention, evidence linking the Clinical Frailty Scale (CFS) to colonic transit time (CTT) remains scarce. In this retrospective study, we examined this association using colon capsule endoscopy (CCE). *Materials and Methods*: We enrolled 124 older adults (64 men, 60 women) aged ≥ 65 years (mean age 74.6 ± 5.6) who underwent CCE for colorectal polyp screening at Aishinkai Nakae Hospital, Japan, between January 2014 and July 2024. CTT was determined at the time of CCE, and frailty was assessed with the Japanese version of the CFS. To avoid the oversimplification inherent in a strict dichotomy, participants were analyzed primarily as three ordered categories—Robust (CFS 1–2; *n* = 73), Intermediate (CFS 3–4; *n* = 43), and Frail (CFS 5–7; *n* = 8)—and, for comparability with previous reports, also as a Robust versus Non-robust (CFS 3–7; *n* = 51) dichotomy. The independent association between CFS and CTT was examined with three complementary multivariable linear regression models (Model 2: CFS continuous; Model 3: CFS three-category ordinal; Model 3b: CFS three-category dummy with Robust as reference), each adjusted for 10 covariates: age, sex, body mass index, type 2 diabetes mellitus, laxative use, anticholinergic drug use, psychotropic drug use, hypothyroidism, smoking history, and history of abdominal surgery. *Results*: CTT rose stepwise across the three frailty categories (Robust: 132.0 min [IQR 81.0–229.0], 157.9 ± 99.9; Intermediate: 198.0 min [90.5–267.0], 186.1 ± 99.8; Frail: 241.5 min [199.0–320.5], 295.8 ± 181.3; Kruskal–Wallis *p* = 0.027; Jonckheere–Terpstra *P* for trend = 0.017); the dichotomous comparison was concordant (median 214.0 vs. 132.0 min; Mann–Whitney U *U* = 1441.5; *p* = 0.033). A graded dose–response-like relationship was further supported by Spearman correlation between the CFS score and CTT (*ρ* = 0.232; *p* = 0.009). Women had significantly longer CTT than men (median 189 vs. 121 min; *p* = 0.013), and the three-group trend was significant within the female subgroup (Kruskal–Wallis *p* = 0.032) but not within men (*p* = 0.138). In multivariable analysis, CFS remained independently associated with CTT prolongation both as a continuous score (Model 2: *β* = 36.6 per 1-point increase; 95% CI 17.3 to 56.0; *p* < 0.001) and as an ordinal three-category predictor (Model 3: *β* = 52.7 per one-step advance; 95% CI 19.7 to 85.8; *p* = 0.002). In the dummy-coded specification (Model 3b), the Frail-versus-Robust contrast was markedly significant (*β* = 155.4 min; 95% CI 71.5 to 239.3; *p* < 0.001), whereas the Intermediate-versus-Robust contrast was not (*β* = 27.0; 95% CI −15.5 to 69.5; *p* = 0.210). Female sex retained independent significance across models (*β* ≈ 47–49 min; *p* = 0.016–0.021). The original dichotomous Non-robust versus Robust specification (Model 1) was no longer significant after full adjustment (*p* = 0.372). *Conclusions*: Frailty progression, as assessed by the CFS, is independently and monotonically associated with prolonged CTT in older adults, with the effect becoming statistically manifest predominantly at the more advanced end of the frailty spectrum and particularly in women. Although these findings do not establish causality between frailty and constipation—and frailty itself reflects a multifactorial age-related process that may not be fully reversible—CCE-measured CTT provides an objective, radiation-free physiological biomarker of frailty-related bowel dysfunction that may support risk stratification and inform future interventional trials targeting potentially modifiable components of the gut–muscle axis.

## 1. Introduction

Managing the health of older adults involves a wide range of interlocking challenges. Among these, frailty—a state of increased vulnerability to stressors arising from an age-related decline in physiological reserve—has received considerable attention as a risk factor for functional dependence and mortality [1,2,3,4,5,6]. The Clinical Frailty Scale (CFS) is globally recognized as a simple yet comprehensive tool for assessing frailty and is widely used in everyday clinical practice [7,8]. In Japan, both the reliability and the validity of the Japanese version of the CFS have been confirmed, and its clinical use has continued to expand [9,10,11]. Its relationship to specific organ-system dysfunctions, however, particularly gastrointestinal motility, remains insufficiently characterized.

Constipation is one of the most common gastrointestinal complaints in older adults and can substantially impair health-related quality of life [12,13,14,15,16]. Its etiology is multifactorial, involving reduced colonic peristalsis, autonomic nervous system dysfunction, and the effects of multiple medications, and prolonged colonic transit time (CTT) plays a central role in its pathophysiology [16,17,18,19]. CTT has traditionally been measured using radiopaque marker methods [20], but colon capsule endoscopy (CCE) has more recently enabled a non-invasive assessment of CTT [21,22,23,24,25]. By recording the transit of an ingested capsule camera through the gastrointestinal tract, CCE allows objective measurement of CTT and is particularly suitable for older adults with multiple comorbidities in whom more invasive examinations may not be feasible [26,27,28].

Several mechanisms have been proposed to explain prolonged CTT in frail older adults, including reduced physical activity, weakened abdominal musculature due to sarcopenia, drug-induced constipation arising from polypharmacy, and hypothyroidism [29,30,31,32,33]. Direct reports examining the association between CFS-defined frailty severity and CTT, however, remain scarce [34].

The present study therefore aimed to examine the association between the CFS score and CTT in older adults undergoing CCE, and to determine whether frailty is independently associated with delayed colonic transit after comprehensive adjustment for confounders. We did not set out to demonstrate that frailty causes constipation, nor that improving frailty would alleviate it; rather, our goal was to establish whether CCE-measured CTT can serve as an objective physiological marker of frailty-related colonic dysfunction in older adults, thereby providing a quantitative substrate on which future interventional studies of the gut–muscle axis could be designed.

## 2. Materials and Methods

### 2.1. Study Design and Population

This single-center retrospective study was conducted at Aishinkai Nakae Hospital in Wakayama, Japan, between January 2014 and July 2024. Consecutive patients aged ≥ 65 years who underwent CCE at the hospital’s Endoscopy Center for colorectal polyp screening were enrolled. The exclusion criteria were as follows: (1) incomplete CCE examination; (2) missing CFS assessment data; (3) a history of inflammatory bowel disease; (4) history of colonic resection; and (5) insufficient medical record documentation precluding the evaluation of confounders affecting intestinal motility.

The following background variables were recorded for each patient: age, sex, body mass index (BMI), presence of type 2 diabetes mellitus, laxative use, anticholinergic drug use, psychotropic (hypnotic) drug use, hypothyroidism, smoking history, and history of abdominal surgery. For the purposes of this study, ‘history of abdominal surgery’ was defined inclusively as any prior surgical procedure involving the abdominal or pelvic cavity, encompassing hysterectomy, cholecystectomy, appendectomy, gastric or upper gastrointestinal surgery, and other pelvic gynecological operations, in view of the potential for adhesion formation or altered bowel motility regardless of the primary operative field. In the present cohort, no patient had a history of cesarean section; the gynecological/pelvic operations recorded were surgery for ectopic pregnancy (*n* = 1), uterine (cervical/endometrial) cancer (*n* = 1), and endometriosis (*n* = 1). Patients with a history of colonic resection were excluded a priori (Section 2.1, exclusion criterion 4). Laxative use was defined as the regular intake (≥3 days/week for ≥4 consecutive weeks) of a physician-prescribed laxative at the time of CCE. By mechanism of action, laxatives were classified into osmotic agents (magnesium oxide, polyethylene glycol), stimulant agents (sennoside, bisacodyl, sodium picosulfate, senna preparations such as Alosenn), and bulk-forming agents; secretagogues (lubiprostone, linaclotide) and ileal bile acid transporter (IBAT) inhibitors (elobixibat) were excluded from this operational definition because they act through distinct mechanisms and, importantly, no patient in the present cohort was receiving such agents. The laxatives actually prescribed at the time of CCE were magnesium oxide (an osmotic agent; *n* = 20), sennoside (a stimulant agent; *n* = 8), and a senna preparation (Alosenn; a stimulant agent; *n* = 1); thus, all 29 laxative users in this cohort were receiving either an osmotic or a conventional stimulant laxative. BMI was included as a potential confounder, given its established association with both frailty and colonic motility.

### 2.2. Ethical Considerations

This study was approved by the Institutional Review Board of Aishinkai Nakae Hospital (approval number 035; approval date 21 September 2025) and conducted in accordance with the principles of the Declaration of Helsinki. Given the retrospective nature of the study, the requirement for written informed consent was waived by the Institutional Review Board.

### 2.3. Frailty Assessment

Frailty was assessed using the Japanese version of the CFS. The CFS is scored on a 9-point scale ranging from 1 (very fit) to 9 (terminally ill); in the present study, the observed CFS scores ranged from 1 to 7. The distribution of scores was as follows: CFS 1, *n* = 2; CFS 2, *n* = 71; CFS 3, *n* = 39; CFS 4, *n* = 4; CFS 5, *n* = 2; CFS 6, *n* = 5; and CFS 7, *n* = 1. To avoid the oversimplification inherent in a strict dichotomy and to better capture the frailty continuum, frailty status was analyzed using two complementary classifications. First, a three-category ordinal classification was applied: Robust (CFS 1–2; *n* = 73), Intermediate (CFS 3–4; *n* = 43; individuals with some activity limitation but who remain independent in activities of daily living), and Frail (CFS 5–7; *n* = 8; individuals requiring assistance with instrumental or basic activities of daily living). Second, for comparability with previously published studies, a dichotomous classification into Robust (CFS 1–2; *n* = 73) and Non-robust (CFS 3–7; *n* = 51) was also applied, on the basis of whether the participant retained activity levels sufficient to influence intestinal peristalsis. CFS assessments were performed by trained medical staff prior to CCE. The nine categories of the CFS are defined as follows: CFS 1 (very fit), robust, active, energetic, and motivated, and regularly exercises; CFS 2 (well), active but not regularly very active; CFS 3 (managing well), medical problems are well controlled but not regularly active beyond routine walking; CFS 4 (vulnerable), not dependent on others for daily help, but symptoms often limit activities; CFS 5 (mildly frail), more evident slowing, and help needed with high-order instrumental activities of daily living; CFS 6 (moderately frail), help needed with all outside activities and with keeping house, and difficulty with stairs; CFS 7 (severely frail), completely dependent on others for personal care; CFS 8 (very severely frail), completely dependent and approaching the end of life; CFS 9 (terminally ill), approaching the end of life with a life expectancy of <6 months.

### 2.4. Measurement of Colonic Transit Time Using Colon Capsule Endoscopy

The PillCam COLON 2 capsule (Medtronic, Inc., Minneapolis, MN, USA) was used for CCE. Bowel preparation and the examination protocol followed the regimen recommended by the Japanese Association for Capsule Endoscopy (JACE) [25,26,35]. The complete preparation schedule, with explicit timing, was as follows: (i) Day −1 (the day before examination): patients consumed a low-residue diet from breakfast through dinner, with no restriction on fluids. (ii) Day −1, 21:00–22:00 (approximately 12 h before capsule ingestion): 50 mg of magnesium citrate dissolved in 180 mL of water was administered as a pre-procedure osmotic laxative. (iii) Day −1, at bedtime (≈23:00): 10 mg of 0.75% sodium picosulfate dissolved in 80 mL of water was administered. (iv) Day 0 (examination day), starting at 08:00: 500–1000 mg of ascorbic acid-containing polyethylene glycol (PEG) solution was administered with 250–500 mL of water and continued until the rectal effluent became clear; the mean duration of preparation on Day 0 before capsule ingestion was approximately 2–3 h. (v) Immediately after the effluent became clear (typically 10:00–11:00), the capsule was ingested together with 20 mg of mosapride citrate. (vi) Booster 1 was administered 1 h after confirmed entry of the capsule into the small bowel (i.e., approximately 60 min after ingestion): 30 mg castor oil combined with 100 mL of ascorbic acid-containing PEG solution. (vii) Booster 2 was administered 3 h after capsule ingestion: 400 mg ascorbic acid-containing PEG solution with 250 mL of water. (viii) Booster 3 was administered when the capsule had not reached the cecum by 5 h after ingestion: 500 mg ascorbic acid-containing PEG solution with 250 mL of water. (ix) Rescue interventions were implemented when the capsule failed to advance—defined as remaining in the stomach beyond 1 h after ingestion, remaining in the same small-bowel segment for more than 2 h on real-time monitoring, or failing to be completely excreted within 8 h of ingestion. Rescue measures were applied in a stepwise manner: first, intramuscular administration of 10 mg metoclopramide for delayed gastric emptying; second, oral administration of 30 mg castor oil in 100 mL of water; third, oral administration of 50 mg magnesium citrate in 180 mL of water; and finally, a 60 mg glycerin enema if the capsule had reached the rectum without spontaneous expulsion. The protocol was discontinued and the examination considered incomplete if the capsule had not been expelled within 10 h of ingestion. CTT was defined as the time (in minutes) from the capsule’s cecal arrival to its expulsion from the anus and was calculated using dedicated software (version 8.0 and 8.3). CCE videos were reviewed by two expert physicians (NK and KM), who were blinded to patients’ CFS scores to minimize bias in CTT measurement.

### 2.5. Study Endpoints

The primary endpoint was the comparison of CTT across the three frailty categories (Robust [CFS 1–2], Intermediate [CFS 3–4], and Frail [CFS 5–7]); the dichotomous Robust versus Non-robust contrast was retained as a secondary endpoint for comparability with the prior literature. Additional secondary endpoints were the Spearman correlation between the CFS score (as a continuous variable) and CTT, and the identification of independent factors associated with CTT prolongation through the three multivariable regression specifications.

### 2.6. Statistical Analysis

Continuous variables are presented as median [interquartile range (IQR)]—given the non-normal distributions observed for CTT and BMI (Shapiro–Wilk test, *p* < 0.001)—or as mean ± standard deviation (SD) when normally distributed. Categorical variables are expressed as frequencies (*n*, %).

For comparisons between the Robust and Non-robust groups, the Mann–Whitney U test was used for continuous variables (age, CTT, and BMI), as the Shapiro–Wilk test indicated non-normal distributions for CTT (Robust group, *p* < 0.001; Non-robust group, *p* < 0.001) and BMI (Robust group, *p* < 0.001). Between-group differences in continuous variables across the three frailty categories were evaluated using the Kruskal–Wallis test, and a monotonic trend across the ordered categories was assessed using the Jonckheere–Terpstra test. The chi-square test or Fisher’s exact test (when any expected frequency was below 5) was used for between-group comparisons of categorical variables.

Spearman’s rank correlation coefficient was used to assess the correlation between the CFS score (as a continuous variable) and CTT. To visualize the stepwise association between the two, the mean CTT and its distribution were plotted for each CFS category.

For the multivariable analysis, multiple linear regression was performed with CTT as the (continuous) dependent variable. Three complementary specifications of frailty were prespecified. The primary model (Model 2) entered the CFS score as a continuous variable. A sensitivity model (Model 3) entered the three-category CFS classification as an ordinal predictor (Robust = 0, Intermediate = 1, Frail = 2). A second sensitivity model (Model 3b) entered the three-category classification as two dummy variables with Robust as the reference, yielding direct Intermediate-versus-Robust and Frail-versus-Robust contrasts. For historical comparability, the original dichotomous specification (Model 1: Non-robust [CFS 3–7] vs. Robust [CFS 1–2]) was also fitted. Each model adjusted for the same set of 10 covariates: age, sex (female), BMI, type 2 diabetes mellitus, laxative use, anticholinergic drug use, psychotropic (hypnotic) drug use, hypothyroidism, smoking history, and history of abdominal surgery. To minimize potential multicollinearity between age and BMI, both variables were mean-centered before model entry. Multicollinearity was assessed using the variance inflation factor (VIF), with VIF < 5 defined as the threshold for the absence of multicollinearity.

All statistical analyses were performed using IBM SPSS Statistics version 31.0.1.0 (IBM Corp., Armonk, NY, USA). Statistical significance was defined as *p* < 0.05 (two-tailed).

## 3. Results

A total of 124 consecutive patients aged ≥ 65 years (64 males, 60 females; median age 74.0 years [IQR 70.0–78.0]; median BMI 22.6 kg/m^2^ [IQR 20.6–23.5]) were enrolled. The CTT for the entire cohort had a median of 155 min (IQR 86.5–246.0; range 14–677 min). Patients were classified primarily into three ordered frailty categories—Robust (CFS 1–2; *n* = 73), Intermediate (CFS 3–4; *n* = 43), and Frail (CFS 5–7; *n* = 8)—and, secondarily, into the conventional Robust (CFS 1–2) versus Non-robust (CFS 3–7; *n* = 51) dichotomy.

Baseline patient characteristics across the three frailty categories—Robust (CFS 1–2; *n* = 73), Intermediate (CFS 3–4; *n* = 43), and Frail (CFS 5–7; *n* = 8)—are shown in Table 1. Age rose stepwise across the three groups (median 73.0 vs. 76.0 vs. 79.0 years; Kruskal–Wallis *p* = 0.004; Jonckheere–Terpstra *P* for trend = 0.005). The proportions of laxative users and of patients receiving psychotropic (hypnotic) medication tended to increase across the frailty spectrum, although these between-group differences did not reach statistical significance on the categorical tests. For comparability with prior reports, the conventional dichotomous classification (Robust [CFS 1–2; *n* = 73] vs. Non-robust [CFS 3–7; *n* = 51]) is also retained, and the corresponding age difference between these two groups was likewise statistically significant.

Colonic transit time (CTT) increased monotonically across the three frailty categories: the median was 132.0 min (IQR 81.0–229.0) in the Robust group, 198.0 min (IQR 90.5–267.0) in the Intermediate group, and 241.5 min (IQR 199.0–320.5) in the Frail group (means ± SD: 157.9 ± 99.9, 186.1 ± 99.8, and 295.8 ± 181.3 min, respectively). The Kruskal–Wallis test indicated a significant overall between-group difference (*H* = 7.24; *p* = 0.027), and the Jonckheere–Terpstra test confirmed a significant monotonic trend of increasing CTT with advancing frailty (*Z* = 2.39; *P* for trend = 0.017). The conventional Robust versus Non-robust dichotomy yielded a concordant result (median 214.0 vs. 132.0 min; Mann–Whitney U test *U* = 1441.5; *p* = 0.033).

### 3.1. Stepwise Prolongation of CTT with Increasing Frailty Severity (Continuous and Three-Category Analyses)

**Figure 1:**
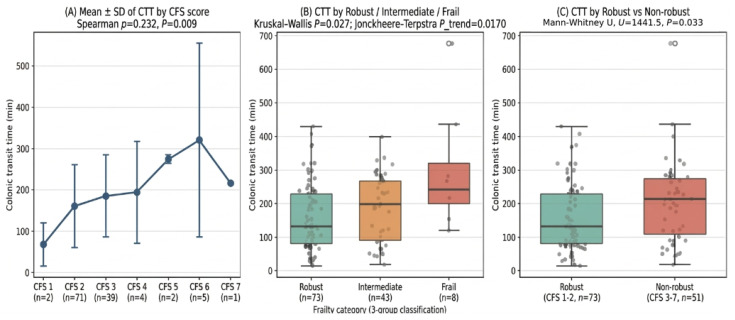
Colonic transit time (CTT) by frailty status (*n* = 124). Panel (**A**): Mean ± SD of CTT plotted against each of the seven individual Clinical Frailty Scale (CFS) scores observed in the cohort, with Spearman’s rank correlation between the CFS score and CTT (*ρ* = 0.232; *p* = 0.009). Panel (**B**): Distribution of CTT across the three-category ordinal classification—Robust (CFS 1–2; *n* = 73), Intermediate (CFS 3–4; *n* = 43), and Frail (CFS 5–7; *n* = 8)—shown as box-and-whisker plots with overlaid individual data points (box, interquartile range; horizontal line, median; whiskers, 1.5 × IQR; outliers, open circles); Kruskal–Wallis test for overall between-group difference (*p* = 0.027) and Jonckheere–Terpstra test for monotonic trend across the ordered categories (*P* for trend = 0.017). Panel (**C**): Distribution of CTT for the conventional dichotomous classification—Robust (CFS 1–2; *n* = 73) vs. Non-robust (CFS 3–7; *n* = 51)—displayed in the same box-and-whisker format, with the Mann–Whitney U test (*U* = 1441.5; *p* = 0.033), retained for comparability with the prior literature. Open circles (**○**) represent outliers beyond 1.5 × IQR from the box edges.

Spearman’s rank correlation, treating the CFS score as a continuous variable, was *ρ* = 0.232 (*p* = 0.009, *n* = 124), indicating a significant positive association between the CFS score and CTT. The three-category analysis was fully concordant: median CTT rose stepwise from the Robust to the Intermediate to the Frail group (132.0 → 198.0 → 241.5 min), supporting a graded dose–response-like relationship across the frailty spectrum (Figure 1A,B).

Sex-stratified analysis showed that females had significantly longer CTT than males (median 189 min [IQR 115.0–271.2] vs. 121 min [IQR 74.8–226.8]; mean 200.2 ± 113.8 vs. 154.5 ± 104.0 min; U = 2420.0; *p* = 0.013). The three-category trend in CTT was significant within the female subgroup (Robust: median 150 min [IQR 99.5–241.0], *n* = 35; Intermediate: 233 min [IQR 177.5–284.0], *n* = 22; Frail: 282 min [IQR 218.0–479.5], *n* = 3; Kruskal–Wallis *p* = 0.032) but not within the male subgroup (Robust: 118 min [IQR 74.2–204.2], *n* = 38; Intermediate: 100 min [IQR 64.0–230.0], *n* = 21; Frail: 216 min [IQR 214.0–267.0], *n* = 5; Kruskal–Wallis *p* = 0.138). Within the dichotomous classification, the CTT difference between the Robust and Non-robust groups was likewise significant among females (median 150 vs. 234 min; *U* = 269.5; *p* = 0.012) but not among males (median 118 vs. 158.5 min; U = 443.0; *p* = 0.490). In the univariable analysis, none of the additional potential confounders—anticholinergic drug use, psychotropic (hypnotic) drug use, hypothyroidism, smoking history, or history of abdominal surgery—was significantly associated with CTT (*p* = 0.51–0.99) (Table 1).

### 3.2. Multivariate Linear Regression Analysis

The multivariable linear regression results are presented in Table 2. In the primary model (Model 2), which entered the CFS score as a continuous variable, the maximum VIF across all 11 variables was 1.27, indicating no multicollinearity; the CFS score (*β* = 36.6 per 1-point increase; 95% CI 17.3 to 56.0; *p* < 0.001) and female sex (*β* = 48.5; 95% CI 9.1 to 88.0; *p* = 0.016) emerged as independent factors associated with CTT prolongation (*R*^2^ = 0.164; adjusted *R*^2^ = 0.082; *F* = 1.997; *p* = 0.035). In the sensitivity analysis using the three-category ordinal classification (Model 3; maximum VIF = 1.50), each one-step advance in frailty category was independently associated with a 52.7 min increase in CTT (*β* = 52.7; 95% CI 19.7 to 85.8; *p* = 0.002), and female sex retained its independent association (*β* = 47.4; 95% CI 7.3 to 87.5; *p* = 0.021). When the three groups were entered as dummy variables with Robust as the reference (Model 3b), the Frail group showed a markedly prolonged CTT relative to the Robust group (*β* = 155.4 min; 95% CI 71.5 to 239.3; *p* < 0.001), whereas the Intermediate group did not differ significantly from the Robust group (*β* = 27.0; 95% CI −15.5 to 69.5; *p* = 0.210); female sex again remained an independent factor (*β* = 48.0; 95% CI 8.3 to 87.7; *p* = 0.018). Taken together, the three regression specifications converged in showing that frailty severity is independently and monotonically associated with CTT prolongation, and that the effect becomes statistically manifest predominantly at the more advanced end of the frailty spectrum. For completeness, the original dichotomous specification (Model 1: Non-robust vs. Robust) is also reported (*R*^2^ = 0.097; adjusted *R*^2^ = 0.008; *F* = 1.09; *p* = 0.372); it was no longer statistically significant in the fully adjusted model, underscoring the importance of incorporating the ordinal structure of the CFS.

Dependent variable: colonic transit time (min), *n* = 124. Each model additionally includes 10 covariates (age, female sex, BMI, type 2 diabetes mellitus, laxative use, anticholinergic drug use, psychotropic [hypnotic] drug use, hypothyroidism, smoking history, and history of abdominal surgery; in the present cohort, the latter encompassed hysterectomy, cholecystectomy, appendectomy, gastric/upper-GI surgery, and pelvic gynecological surgery for ectopic pregnancy, uterine cancer or endometriosis—no patient had a history of cesarean section).

Model 2 (primary): *R*^2^ = 0.164; adjusted *R*^2^ = 0.082; *F* = 1.997; *P*(*F*) = 0.035; maximum VIF = 1.27. Model 3 (ordinal, sensitivity): *R*^2^ = 0.136; adjusted *R*^2^ = 0.051; *F* = 1.60; *P*(*F*) = 0.107; maximum VIF = 1.50. Model 3b (dummy, sensitivity): *R*^2^ = 0.163; adjusted *R*^2^ = 0.072; F = 1.78; *P*(*F*) = 0.057. Model 1 (Robust vs. Non-robust dichotomous, retained for historical comparison): *R*^2^ = 0.097; adjusted *R^2^* = 0.008; *F* = 1.09; *P*(*F*) = 0.372.

## 4. Discussion

The principal finding of this study is that CTT, measured by CCE, is independently and monotonically associated with frailty severity assessed by the CFS. Across the three frailty categories (Robust, Intermediate, Frail), the median CTT rose stepwise from 132.0 to 198.0 to 241.5 min (Kruskal–Wallis *p* = 0.027; Jonckheere–Terpstra *P* for trend = 0.017). The Frail group showed a markedly prolonged CTT compared with the Robust group (adjusted *β* = 155.4 min; 95% CI 71.5 to 239.3; *p* < 0.001; Model 3b), whereas the Intermediate group did not differ significantly after full adjustment (*p* = 0.210). The conventional dichotomous comparison (Robust vs. Non-robust) also showed prolonged CTT in the Non-robust group (median 214 vs. 132 min; *p* = 0.033).

In multivariable linear regression, the CFS—whether modeled as a continuous score (*β* = 36.6 per 1-point increase; *p* < 0.001) or as a three-category ordinal predictor (*β* = 52.7 per category step; *p* = 0.002)—and female sex (*β* ≈ 48 min; *p* = 0.016–0.021) emerged as the only independent determinants of CTT after simultaneous adjustment for ten additional covariates. The dummy-coded sensitivity analysis (Model 3b) localized this independent effect predominantly to the advanced end of the frailty spectrum. Notably, thedichotomous Robust versus Non-robust contrast (Model 1) was no longer significant after full adjustment (*p* = 0.372), underscoring the analytical value of preserving the ordinal structure of the CFS rather than collapsing it into a single binary contrast.

Although age increased monotonically across the three frailty categories (73.0 → 76.0 → 79.0 years; *P* for trend = 0.005), it was not retained as an independent factor in any of the multivariable specifications (all *p* > 0.6). This suggests that frailty status—rather than chronological age per se—is the more proximal correlate of delayed colonic transit in older adults. Type 2 diabetes mellitus and laxative use likewise showed no significant association with CTT, plausibly reflecting the limited number of diabetic patients (19/124), the modest number of laxative users (*n* = 29; all on osmotic or stimulant agents), and the homogeneous bowel preparation achieved under the standardized JACE protocol.

### 4.1. Feasibility of CCE-Based CTT Measurement and Artificial Intelligence Applications

The conventional radiopaque marker method, scintigraphy, and the wireless motility capsule all provide complementary information on colonic transit [36], but each entails either radiation exposure or specialized equipment. By contrast, CCE simultaneously captures intraluminal video imaging and yields a quantitative CTT, and its measurement and reporting have been standardized in the recent ESGE Nyborg consensus [21]. The procedure is also pharmacologically modifiable: Deding et al. showed that adjunctive prucalopride improves CCE completion rates to 74.9% and shortens CTT by more than 2 h on average [37]. Persistent challenges—including the complexity of bowel preparation, cost, variable completion rates, and the expertise required for image interpretation—remain [23]; nevertheless, the radiation-free and minimally invasive nature of CCE makes it particularly attractive for the physiological assessment of intestinal function in frail older adults.

Building on this technical platform, the integration of artificial intelligence (AI) into the analysis of CCE-derived data represents a logical next step. Deep-learning models can already enable automated, observer-independent quantification of CTT from CCE footage, substantially reducing the interpretive workload [38,39], while machine-learning frameworks that combine the CFS score with gut microbiota profiles, dietary and physical-activity data, and medication exposure could support the personalized identification of frail older adults at greatest risk of delayed colonic transit [40,41]. Prospective validation studies linking AI-based CTT measurement with frailty outcomes will be an important next step.

### 4.2. Comparison and Integration of CFS with Objective Frailty Measures

Although we used the CFS as a pragmatic clinician-rated instrument, other validated frailty constructs—most notably the Short Physical Performance Battery (SPPB) and the modified Fried phenotype—rely on objective physical measures. A 2024 validation study reported a very strong inverse correlation between the CFS and SPPB (*r* = −0.838, *p* < 0.001) and a strong correlation between the CFS and the modified Fried criteria (*r* = −0.725, *p* < 0.001) [42]. Crosswalks across frailty scales have likewise shown substantial concordance among the CFS, the frailty phenotype, and the FRAIL scale [43], and systematic reviews support the use of simple tools such as the CFS for screening, with multidimensional instruments reserved for more detailed characterization [44]. Future studies should therefore combine the CFS with objective measures such as the SPPB and handgrip strength—a validated surrogate for muscle strength [45]—to clarify which dimensions of frailty most directly drive prolonged CTT.

### 4.3. Strategies for Improving Colonic Transit Time and Overall Health, Autonomy

Pharmacologically, the 5-HT4 receptor agonist prucalopride reduces CTT by 12–14 h in pooled analyses [46] and is effective irrespective of age, BMI, or renal function [47]. Elobixibat improves rectal sensory thresholds in patients aged ≥ 60 years [48], and lubiprostone, linaclotide, and elobixibat have all been shown to be effective for chronic constipation in adults [49]. Consensus reviews accordingly recommend a stepwise combination of fiber optimization, osmotic laxatives, and—where indicated—newer prokinetic or secretagogue agents and probiotics, particularly in older patients [50,51].

Non-pharmacologically, even brief periods of physical inactivity prolong total CTT from 10.9 to 19.5 h in previously active older individuals [52], while higher habitual physical activity is consistently associated with shorter colonic transit [33]. Abdominal massage (10 min sessions, 5 days/week, for 4 weeks) improves stool consistency, abdominal bloating, stool volume, and defecation frequency in older adults [53], and recent reviews confirm consistent reductions in constipation severity and CTT across randomized trials [54]. Multi-strain probiotics significantly increase stool frequency (MD = 1.02; 95% CI 0.21–1.83; *p* < 0.05) and ameliorate constipation-related symptoms in older patients [55]. Oral-function and nutritional interventions [56,57], together with aerobic exercise and adequate fluid intake [58], represent additional rational components of comprehensive care. Taken together, these observations support the design of multicenter prospective trials evaluating multimodal programmes that combine individualized exercise, dietary fiber and protein optimization, hydration, abdominal massage, probiotics, and—where appropriate—prokinetic agents, with CCE-measured CTT serving as a sensitive physiological co-primary endpoint alongside validated frailty and functional-autonomy outcomes.

Frailty is a multidimensional process that affects overall health, functional autonomy, and colonic motility in parallel, and these three domains are linked through a bidirectional gut–muscle axis: sarcopenia, malnutrition, and physical inactivity may prolong CTT, while impaired colonic motility, dysbiosis, and reduced nutrient absorption may in turn accelerate the frailty trajectory [31,59]. Because the present design is cross-sectional and retrospective, causality cannot be established, and the underlying age-related decline that defines frailty is unlikely to be fully reversed by any external intervention. Within these limits, however, the available evidence supports parallel intervention across all three domains rather than the isolated treatment of constipation.

### 4.4. Strengths and Limitations

The principal methodological strengths of this study are the systematic adjustment for 11 potential confounders entered simultaneously into each regression model, the application of a standardized JACE-recommended CCE preparation protocol to all participants, and the use of three complementary specifications of frailty (continuous CFS, three-category ordinal CFS, and dichotomous Robust/Non-robust). Across all three specifications, the CFS-based predictor and female sex consistently emerged as the only independent determinants of prolonged CTT, whereas the simple dichotomous Robust versus Non-robust contrast lost significance after full adjustment (*p* = 0.372). This pattern reveals a graded, dose–response-like relationship between frailty severity and CTT—most pronounced at the Frail end of the spectrum (adjusted *β* = 155.4 min vs. Robust; *p* < 0.001)—and underscores the value of preserving the ordinal structure of the CFS rather than collapsing it into a single binary variable.

Several limitations warrant acknowledgment. First, the single-center retrospective design raises concerns about selection bias and external validity. Second, the sample size was modest (*n* = 124), with only eight patients in the most severe (CFS 5–7) stratum, constraining the resolution of stepwise contrasts. Third, the explanatory power of the regression models was modest, indicating that CTT variability is shaped by additional factors—including dietary content, daily physical activity, gut microbiota composition, and autonomic function—that were not captured here. Fourth, CTT values measured by CCE inevitably reflect the influence of bowel preparation. Fifth, residual confounding from sarcopenia severity, opioid use, and detailed dietary factors cannot be excluded. Sixth, BMI-related findings in predominantly Western cohorts—such as Liu et al.’s NHANES analysis [59]—may not translate directly to the present Japanese sample, in which BMI was not an independent determinant of CTT after multivariable adjustment. Seventh, bowel-movement frequency was not consistently recorded in the source medical records and could not be analyzed in relation to CTT; correlating subjective stool frequency with objective CTT will be an important task for future prospective studies. Finally, because the design is associational, our data establish an independent association between frailty severity and prolonged CTT but cannot demonstrate causality; whether frailty-directed interventions can shorten CTT must be tested in prospective interventional trials that incorporate standardized recording of stool frequency, dietary intake, physical-activity quantification, sarcopenia indices, and CCE-measured CTT.

## 5. Conclusions

In older adults, the progression of frailty, as assessed by the CFS, is significantly and independently associated with the stepwise prolongation of CTT measured by CCE. The association was consistent across three complementary modelling strategies—continuous CFS, three-category ordinal CFS (Robust/Intermediate/Frail), and dummy-coded contrasts—with the effect becoming statistically manifest predominantly at the more advanced end of the frailty spectrum and particularly in women. Although these associations do not establish causality, they support the use of CCE-measured CTT as an objective, radiation-free, minimally invasive physiological marker of frailty-related colonic dysfunction in older adults. Whether frailty-directed interventions can meaningfully modify CTT—and whether such modification translates into improvements in stool frequency, symptoms, or quality of life—will need to be evaluated in dedicated prospective interventional trials.

## Figures and Tables

**Table 1:** Baseline patient characteristics across the three frailty categories (Robust [CFS 1–2; *n* = 73], Intermediate [CFS 3–4; *n* = 43], and Frail [CFS 5–7; *n* = 8]).

Variable	Robust (CFS 1–2; *n* = 73)	Intermediate (CFS 3–4; *n* = 43)	Frail (CFS 5–7; *n* = 8)	*p*-Value *	*P* for Trend †
Age, years, median [IQR]	73.0 [70.0–77.0]	76.0 [71.0–78.5]	79.0 [76.8–84.8]	0.004	0.005
Female sex, *n* (%)	35 (47.9)	22 (51.2)	3 (37.5)	0.772	0.986
BMI, kg/m^2^, median [IQR]	23.0 [21.2–23.5]	22.2 [19.8–23.6]	22.1 [18.6–23.1]	0.191	0.073
Type 2 diabetes mellitus, *n* (%)	13 (17.8)	5 (11.6)	1 (12.5)	0.654	0.580
Laxative use, *n* (%) ^a^	14 (19.2)	13 (30.2)	2 (25.0)	0.395	0.366
Anticholinergic drug use, *n* (%)	4 (5.5)	1 (2.3)	1 (12.5)	0.433	0.954
Psychotropic (hypnotic) use, *n* (%)	8 (11.0)	10 (23.3)	1 (12.5)	0.201	0.372
Hypothyroidism, *n* (%)	1 (1.4)	1 (2.3)	0 (0.0)	0.862	0.972
Smoking history, *n* (%)	7 (9.6)	4 (9.3)	1 (12.5)	0.961	0.962
History of abdominal surgery, *n* (%) ^b^	10 (13.7)	2 (4.7)	2 (25.0)	0.148	0.712
Colonic transit time, min, median [IQR]	132.0 [81.0–229.0]	198.0 [90.5–267.0]	241.5 [199.0–320.5]	0.027	0.017
Colonic transit time, min, mean ± SD	157.9 ± 99.9	186.1 ± 99.8	295.8 ± 181.3	—	—

* Kruskal–Wallis test for continuous variables; chi-square or Fisher-type test for categorical variables. † Jonckheere–Terpstra trend test for monotonic association across the three ordered frailty categories. ^a^ Laxative use was defined as the regular intake (≥3 days/week for ≥4 consecutive weeks) of a physician-prescribed laxative at the time of colon capsule endoscopy. Laxatives were mechanistically classified into osmotic (magnesium oxide, polyethylene glycol), stimulant (sennoside, bisacodyl, sodium picosulfate, senna preparations [e.g., Alosenn]) and bulk-forming agents; secretagogues (lubiprostone, linaclotide) and IBAT inhibitors (elobixibat) were not used by any enrolled patient and were therefore excluded from this definition. The agents actually prescribed in this cohort were magnesium oxide (*n* = 20), sennoside (*n* = 8), and senna preparation (Alosenn; *n* = 1). ^b^ ‘History of abdominal surgery’ includes hysterectomy, cholecystectomy, appendectomy, gastric/upper-GI surgery, and other pelvic gynecological operations; no patient had a history of cesarean section, and the pelvic operations recorded were surgery for ectopic pregnancy (*n* = 1), uterine cancer (*n* = 1), and endometriosis (*n* = 1). History of colonic resection was an exclusion criterion. BMI, body mass index; CFS, Clinical Frailty Scale; CTT, colonic transit time; IBAT, ileal bile acid transporter; IQR, interquartile range; SD, standard deviation.

**Table 2:** Multivariable linear regression analyses of colonic transit time across three modeling specifications of frailty (Model 2: CFS continuous [primary]; Model 3: CFS three-category ordinal; Model 3b: CFS three-category dummy with Robust as reference).

	Model 2: CFS Continuous (Primary)			Model 3: CFS Ordinal (3-Group Sensitivity)			Model 3b: CFS Dummy (3-Group Sensitivity)		
Variable	*β*	95% CI	*p*-Value	*β*	95% CI	*p*-Value	*β*	95% CI	*p*-Value
CFS—primary frailty predictor (Model 2: per 1-pt; Model 3: per category step; Model 3b: Intermediate vs. Robust)	36.63	17.27 to 56.00	<0.001	52.73	19.68 to 85.77	0.002	26.99	−15.47 to 69.45	0.210
CFS—Frail vs. Robust contrast (Model 3b only)	—	—	—	—	—	—	155.37	71.49 to 239.25	<0.001
Age (mean-centered), years	−0.78	−4.61 to 3.06	0.689	−0.33	−4.20 to 3.54	0.867	−0.9	−4.77 to 2.98	0.647
Female sex (vs. male)	48.54	9.06 to 88.03	0.016	47.4	7.26 to 87.54	0.021	47.95	8.25 to 87.65	0.018
BMI (mean-centered), kg/m^2^	3.07	−3.31 to 9.44	0.342	2.98	−3.50 to 9.47	0.364	2.94	−3.47 to 9.36	0.365
Type 2 diabetes mellitus	−9.43	−63.82 to 44.96	0.732	−6.44	−61.83 to 48.95	0.818	−8.3	−63.11 to 46.51	0.765
Laxative use	14.32	−32.22 to 60.85	0.543	13.2	−34.12 to 60.53	0.582	17.01	−29.96 to 63.98	0.475
Anticholinergic drug use	−6.8	−99.05 to 85.44	0.884	−4.77	−98.62 to 89.07	0.920	−14.89	−108.32 to 78.53	0.753
Psychotropic (hypnotic) use	−12.07	−67.99 to 43.85	0.670	−14.08	−70.94 to 42.79	0.625	−7.34	−64.02 to 49.34	0.798
Hypothyroidism	−38.87	−191.87 to 114.12	0.616	−43.2	−198.68 to 112.28	0.583	−39.23	−193.05 to 114.59	0.614
Smoking history	18.31	−48.06 to 84.67	0.586	9.4	−58.14 to 76.94	0.783	8.26	−58.54 to 75.07	0.807
History of abdominal surgery	−16.07	−77.89 to 45.75	0.608	−8.34	−71.17 to 54.50	0.793	−18.85	−81.97 to 44.27	0.555

Predictor coding: in Model 2, CFS is entered as a continuous variable (1 unit = 1-point increase). In Model 3, the three-category CFS classification is entered as a single ordinal predictor (Robust = 0, Intermediate = 1, Frail = 2; 1 unit = one-step advance in frailty category). In Model 3b, the three categories are entered as two dummy variables with Robust as the reference, providing two contrasts: the first row of Table 2 reports the Intermediate-versus-Robust contrast and the second row reports the Frail-versus-Robust contrast. Age and BMI are mean-centered before model entry. Each model additionally adjusts for 10 covariates. BMI, body mass index; CFS, Clinical Frailty Scale; CI, confidence interval; VIF, variance inflation factor.

## Data Availability

The data presented in this study are available on request from the corresponding author. The data are not publicly available due to ethical restrictions and the need to protect patient privacy.

## Abbreviations

The following abbreviations are used in this manuscript:

AI	Artificial intelligence
BMI	Body mass index
CCE	Colon capsule endoscopy
CFS	Clinical Frailty Scale
CTT	Colonic transit time
JACE	Japanese Association for Capsule Endoscopy
SPPB	Short Physical Performance Battery
VIF	Variance Inflation Factor

## References

[1] Kim D.H., Rockwood K. (2024). Frailty in older adults. N. Engl. J. Med..

[2] Dent E., Hanlon P., Sim M., Jylhävä J., Liu Z., Vetrano D.L., Stolz E., Pérez-Zepeda M.U., Crabtree D.R., Nicholson C. (2023). Recent developments in frailty identification, management, risk factors and prevention: A narrative review of leading journals in geriatrics and gerontology. Ageing Res. Rev..

[3] Clegg A., Young J., Iliffe S., Rikkert M.O., Rockwood K. (2013). Frailty in elderly people. Lancet.

[4] Dent E., Hoogendijk E.O. (2023). Trajectories, transitions, and trends in frailty among older adults: A review. Ann. Geriatr. Med. Res..

[5] Morley J.E., Vellas B., van Kan G.A., Anker S.D., Bauer J.M., Bernabei R., Cesari M., Chumlea W.C., Doehner W., Evans J. (2013). Frailty consensus: A call to action. J. Am. Med. Dir. Assoc..

[6] Dent E., Martin F.C., Bergman H., Woo J., Romero-Ortuno R., Walston J.D. (2019). Management of frailty: Opportunities, challenges, and future directions. Lancet.

[7] Dlima S.D., Hall A., Aminu A.Q., Akpan A., Todd C., Vardy E.R.L.C. (2024). Frailty: A global health challenge in need of local action. BMJ Glob. Health.

[8] O’Caoimh R., Sezgin D., O’Donovan M.R., Molloy D.W., Clegg A., Rockwood K., Liew A. (2021). Prevalence of frailty in 62 countries across the world: A systematic review and meta-analysis of population-level studies. Age Ageing.

[9] Komiya H., Suzuki Y., Watanabe K., Nagae M., Nakashima H., Fujisawa C., Miyahara S., Tajima T., Sakai T., Takeya Y. (2025). Validation of the Japanese version of the Clinical Frailty Scale. Geriatr. Gerontol. Int..

[10] Shimura T., Yamamoto M., Kano S., Kagase A., Kodama A., Koyama Y., Tsuchikane E., Suzuki T., Otsuka T., Kohsaka S. (2017). Impact of the clinical frailty scale on outcomes after transcatheter aortic valve replacement. Circulation.

[11] Yuguchi T., Nakajima K., Takaoka H., Shimokawa T. (2024). Usefulness of clinical frailty scale for comprehensive geriatric assessment of older heart failure patients. Circ. Rep..

[12] Xian X., Wang X., Liu J., Yang H. (2024). Investigation of functional constipation in elderly inpatients and analysis of its influencing factors: A cross-sectional study. Medicine.

[13] Lim J., Park H., Lee H., Lee E., Lee D., Jung H.W., Jang I.Y. (2021). Higher frailty burden in older adults with chronic constipation. BMC Gastroenterol..

[14] Arco S., Saldaña E., Serra-Prat M., Palomera E., Ribas Y., Font S., Clavé P., Mundet L. (2022). Functional constipation in older adults: Prevalence, clinical symptoms and subtypes, association with frailty, and impact on quality of life. Digestion.

[15] Manabe N., Fujita M., Haruma K. (2025). The effectiveness of transabdominal ultrasonography in managing chronic constipation in the elderly, with a focus on the underlying pathological conditions. Diagnostics.

[16] Camilleri M., Ford A.C., Mawe G.M., Dinning P.G., Rao S.S., Chey W.D., Simrén M., Lembo A., Young-Fadok T.M., Chang L. (2017). Chronic constipation. Nat. Rev. Dis. Primers.

[17] Rao S.S.C., Rattanakovit K., Patcharatrakul T. (2016). Diagnosis and management of chronic constipation in adults. Nat. Rev. Gastroenterol. Hepatol..

[18] Mearin F., Lacy B.E., Chang L., Chey W.D., Lembo A.J., Simren M., Spiller R. (2016). Bowel disorders. Gastroenterology.

[19] Dinning P.G., Di Lorenzo C. (2011). Colonic dysmotility in constipation. Best Pract. Res. Clin. Gastroenterol..

[20] Metcalf A.M., Phillips S.F., Zinsmeister A.R., MacCarty R.L., Beart R.W., Wolff B.G. (1987). Simplified assessment of segmental colonic transit. Gastroenterology.

[21] Lei I.I., Koulaouzidis A., Schelde-Olesen B., Turvill J., Valdivia P.C., Rondonotti E., Plevris J.N., Keuchel M., Saurin J.-C., Dray X. (2025). Unifying terminology, reporting, and bowel preparation standards in colon capsule endoscopy: Nyborg Consensus. Endoscopy.

[22] Jalayeri Nia G., Arasaradnam R.P., Koulaouzidis A. (2023). Clinical utility of colon capsule endoscopy: A moving target?. Ther. Adv. Gastroenterol..

[23] Eliakim R., Yassin K., Niv Y., Metzger Y., Lachter J., Gal E., Sapoznikov B., Konikoff F., Leichtmann G., Fireman Z. (2009). Prospective multicenter performance evaluation of the second-generation colon capsule compared with colonoscopy. Endoscopy.

[24] Moen S., Vuik F.E.R., Voortman T., Kuipers E.J., Spaander M.C.W. (2022). Predictors of gastrointestinal transit times in colon capsule endoscopy. Clin. Transl. Gastroenterol..

[25] Otani I., Oka S., Tanaka S., Iio S., Tsuboi A., Kunihara S., Nagasaki N., Chayama K. (2020). Diagnostic Yield of Colon Capsule Endoscopy in Detection of Superficial Colorectal Lesions. Digestion.

[26] Pasha S.F. (2018). Applications of Colon Capsule Endoscopy. Curr. Gastroenterol. Rep..

[27] Spada C., Hassan C., Galmiche J.P., Neuhaus H., Dumonceau J.M., Adler S., Epstein O., Gay G., Pennazio M., Rex D.K. (2012). Colon capsule endoscopy: European Society of Gastrointestinal Endoscopy (ESGE) Guideline. Endoscopy.

[28] Spada C., Pasha S.F., Gross S.A., Leighton J.A., Schnoll-Sussman F., Correale L., González Suárez B., Costamagna G., Hassan C. (2016). Accuracy of first and second-generation colon capsules in endoscopic detection of colorectal polyps: A systematic review and meta-analysis. Clin. Gastroenterol. Hepatol..

[29] Li W., Liu C., Zhang Z., Cai Z., Lv T., Zhang R., Zuo Y., Chen S. (2024). Exploring the top 30 drugs associated with drug-induced constipation based on the FDA adverse event reporting system. Front. Pharmacol..

[30] Satake S., Arai H. (2020). The revised Japanese version of the Cardiovascular Health Study criteria (revised J-CHS criteria). Geriatr. Gerontol. Int..

[31] Bharucha A.E., Lacy B.E. (2020). Mechanisms, evaluation, and management of chronic constipation. Gastroenterology.

[32] Xu G.M., Hu M.X., Li S.Y., Ran X., Zhang H., Ding X.F. (2024). Thyroid disorders and gastrointestinal dysmotility: An old association. Front. Physiol..

[33] Jensen M.M., Pedersen H.E., Clemmensen K.K.B., Ekblond T.S., Ried-Larsen M., Færch K., Brock C., Quist J.S. (2024). Associations Between Physical Activity and Gastrointestinal Transit Times in People with Normal Weight, Overweight, and Obesity. J. Nutr..

[34] Ron Y., Leibovitz A., Monastirski N., Habot B., Segal R. (2002). Colonic transit time in diabetic and nondiabetic long-term care patients. Gerontology.

[35] Ohmiya N., Hotta N., Mitsufuji S., Nakamura M., Omori T., Maeda K., Okuda K., Yatsuya H., Tajiri H. (2019). Multicenter feasibility study of bowel preparation with castor oil for colon capsule endoscopy. Dig. Endosc..

[36] Rao S.S., Camilleri M., Hasler W.L., Maurer A.H., Parkman H.P., Saad R., Scott M.S., Simren M., Soffer E., Szarka L. (2011). Evaluation of gastrointestinal transit in clinical practice: Position paper of the American and European Neurogastroenterology and Motility Societies. Neurogastroenterol. Motil..

[37] Deding U., Kaalby L., Baatrup G., Kobaek-Larsen M., Thygesen M.K., Epstein O., Bjørsum-Meyer T. (2022). The Effect of Prucalopride on the Completion Rate and Polyp Detection Rate of Colon Capsule Endoscopies. Clin. Epidemiol..

[38] Ribeiro T., Afonso J., Ferreira J.P.S., Cardoso H., Andrade P., Parente M.P.L., Jorge R.N., Mascarenhas Saraiva M., Macedo G. (2022). Deep learning and colon capsule endoscopy: Automatic detection of blood and colonic mucosal lesions using a convolutional neural network. Endosc. Int. Open.

[39] Moen S., Vuik F.E.R., Kuipers E.J., Spaander M.C.W. (2022). Artificial intelligence in colon capsule endoscopy-a systematic review. Diagnostics.

[40] Mizuguchi Y., Nakao M., Nagai T., Takahashi Y., Abe T., Kakinoki S., Imagawa S., Matsutani K., Saito T., Takahashi M. (2024). Machine learning-based gait analysis to predict clinical frailty scale in elderly patients with heart failure. Eur. Heart J. Digit. Health.

[41] Sajeev S., Champion S., Maeder A., Gordon S. (2022). Machine learning models for identifying pre-frailty in community dwelling older adults. BMC Geriatr..

[42] Soulis G., Kyriakopoulou E., Leventouri A., Zigkiri E., Efthymiou V., Kentros Z., Koutsouri A. (2024). Pilot Testing of Useful Tools’ Validity for Frailty Assessment in Greece: Translated PRISMA-7 Tool, Modified Fried Criteria and Clinical Frailty Scale. Healthcare.

[43] Sison S.D.M., Shi S.M., Kim K.M., Steinberg N., Jeong S., McCarthy E.P., Kim D.H. (2023). A crosswalk of commonly used frailty scales. J. Am. Geriatr. Soc..

[44] Faller J.W., Pereira D.d.N., de Souza S., Nampo F.K., Orlandi F.d.S., Matumoto S. (2019). Instruments for the detection of frailty syndrome in older adults: A systematic review. PLoS ONE.

[45] Ooi H., Welch C. (2024). Obstacles to the early diagnosis and management of sarcopenia: Current perspectives. Clin. Interv. Aging.

[46] Emmanuel A., Cools M., Vandeplassche L., Kerstens R. (2014). Prucalopride improves bowel function and colonic transit time in patients with chronic constipation: An integrated analysis. Am. J. Gastroenterol..

[47] Lembo A., Staller K., Boules M., Feuerstadt P., Spalding W., Gabriel A., Youssef A., Xie Y., Terreri B., Cash B.D. (2024). Efficacy and safety of prucalopride in patients with chronic idiopathic constipation stratified by age, body mass index, and renal function: A post hoc analysis of phase III and IV, randomized, placebo-controlled clinical studies. Ther. Adv. Gastroenterol..

[48] Manabe N., Umeyama M., Ishizaki S., Ota T., Kuratani S., Katsumata R., Fujita M., Haruma K., Camilleri M. (2023). Elobixibat improves rectal sensation in patients with chronic constipation aged ≥60 years: A randomised placebo-controlled study. BMJ Open Gastroenterol..

[49] Rao S.S., Manabe N., Karasawa Y., Hasebe Y., Nozawa K., Nakajima A., Fukudo S. (2024). Comparative profiles of lubiprostone, linaclotide, and elobixibat for chronic constipation: A systematic literature review with meta-analysis and number needed to treat/harm. BMC Gastroenterol..

[50] Hojo M., Shibuya T., Nagahara A. (2025). Management of Chronic Constipation: A Comprehensive Review. Intern. Med..

[51] Ren Y.P., Chan W.L., Chuah K.H., Kim Y.S., Nakajima A., Mahadeva S., Lee Y.Y., Chua A.S.B., Bai T., Syam A.F. (2025). Bali Chronic Constipation Roundtable Report: Chronic Constipation Management in Asia. J. Neurogastroenterol. Motil..

[52] Bingham S.A., Cummings J.H. (1989). Effect of exercise and physical fitness on large intestinal function. Gastroenterology.

[53] Okgun Alcan A., Kizilkaya Beji N. (2021). The effect of abdominal massage on chronic constipation and constipation quality of life in elderly. Int. J. Caring Sci..

[54] Durga G., Mooventhan A., Gowthami R., Nivethitha L., Manavalan N. (2025). Scientific Evidence-based Effects of Abdominal Massage in People with Constipation: A Narrative Review. Int. J. Ther. Massage Bodyw..

[55] Deng X., Shang X., Zhou L., Li X., Guo K., Xu M., Hou L., Hui X., Li S. (2023). Efficacy and Safety of Probiotics in Geriatric Patients with Constipation: Systematic Review and Meta-Analysis. J. Nutr. Health Aging.

[56] Tullio V., La Spina C., Guadagnino D., Albano G.D., Zerbo S., Argo A. (2023). Nutritional therapy and gastrointestinal motility in elderly patients: Systematic review. Healthcare.

[57] Takeda T., Asaoka D., Kiko H., Kanazawa T., Nomura O., Oki S., Hojo M., Sugano K., Matsuno K., Inoshita H. (2025). The Association Between Severity of Constipation and Oral Frailty Index-8 in the JUSTICE-TOKYO Study: A Cross-Sectional Study. Biomedicines.

[58] Kim S.-J. (2024). Diet, Physical Activity, and Chronic Constipation: Unveiling the Combined Effects for Better Treatment Strategies. J. Neurogastroenterol. Motil..

[59] Liu X., Wang Y., Shen L., Sun Y., Zeng B., Zhu B., Dai F. (2023). Association Between Frailty and Chronic Constipation and Chronic Diarrhea Among American Older Adults: National Health and Nutrition Examination Survey. BMC Geriatr..

